# Supplementary figures and images for: Post-ischemic reorganization of sensory responses in cerebral cortex
Source: Front Neurosci. 2023 Jun 2;17:1151309. doi: 10.3389/fnins.2023.1151309 (PMC10272353; doi:10.3389/fnins.2023.1151309)

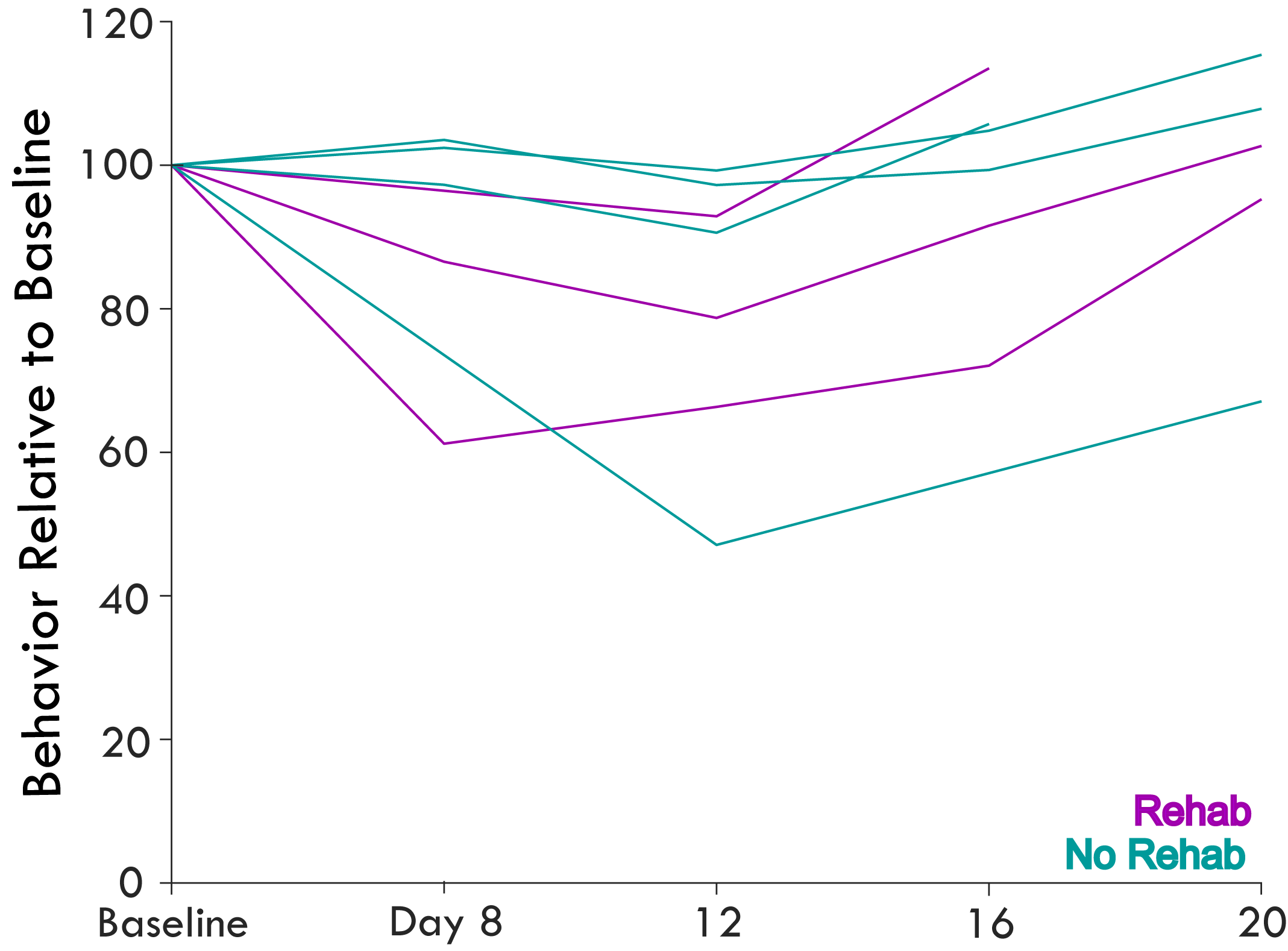

Supplement: Supplementary file 1 [file Image_1.TIF]

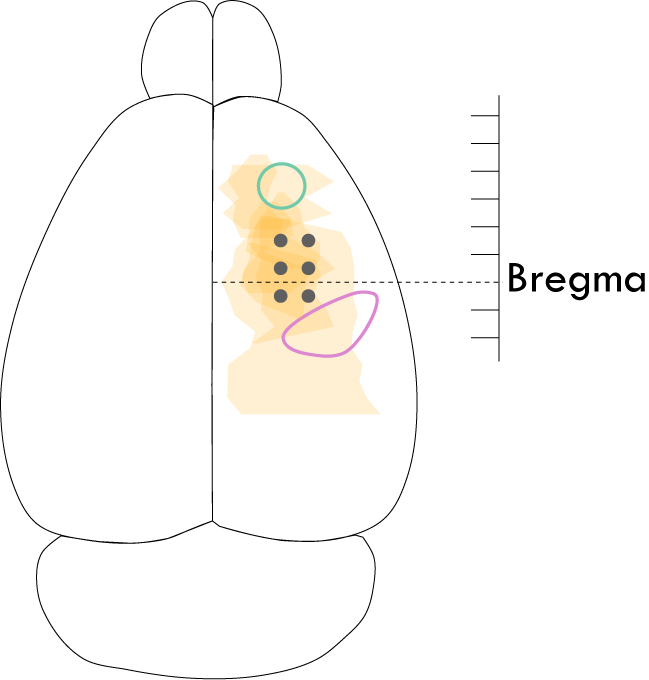

Supplement: Supplementary file 2 [file Image_2.TIF]

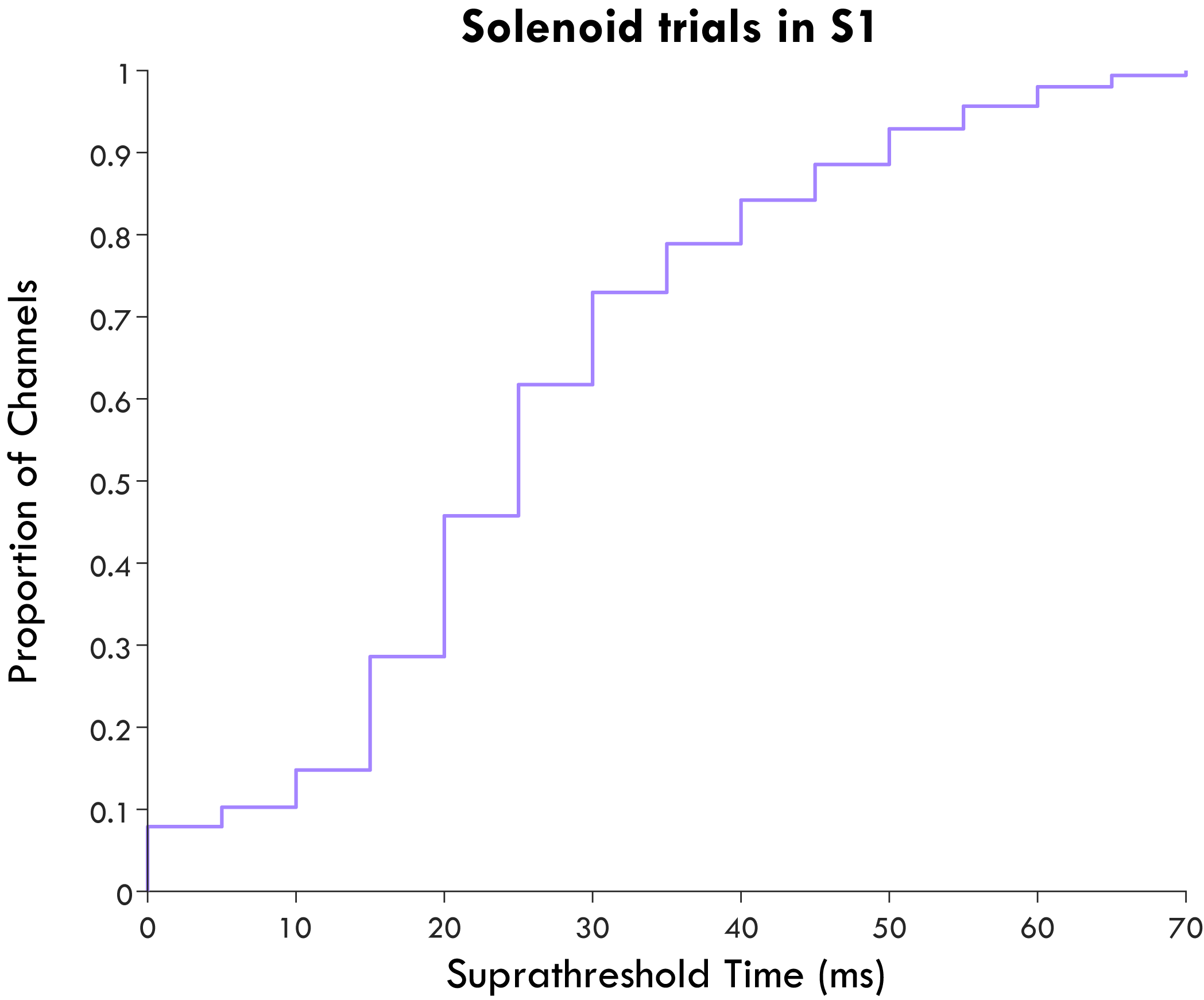

Supplement: Supplementary file 3 [file Image_3.TIF]

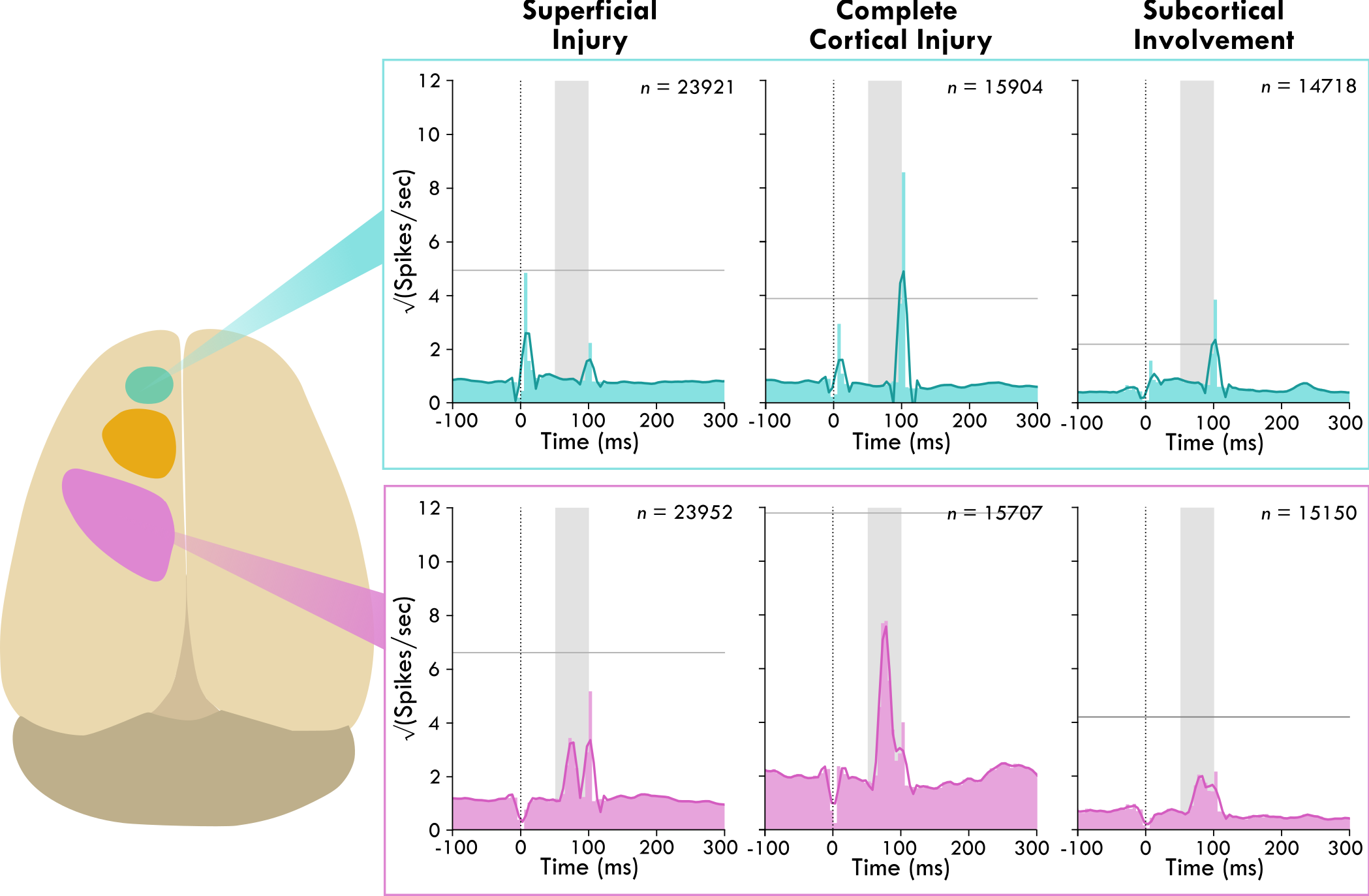

Supplement: Supplementary file 4 [file Image_4.TIF]

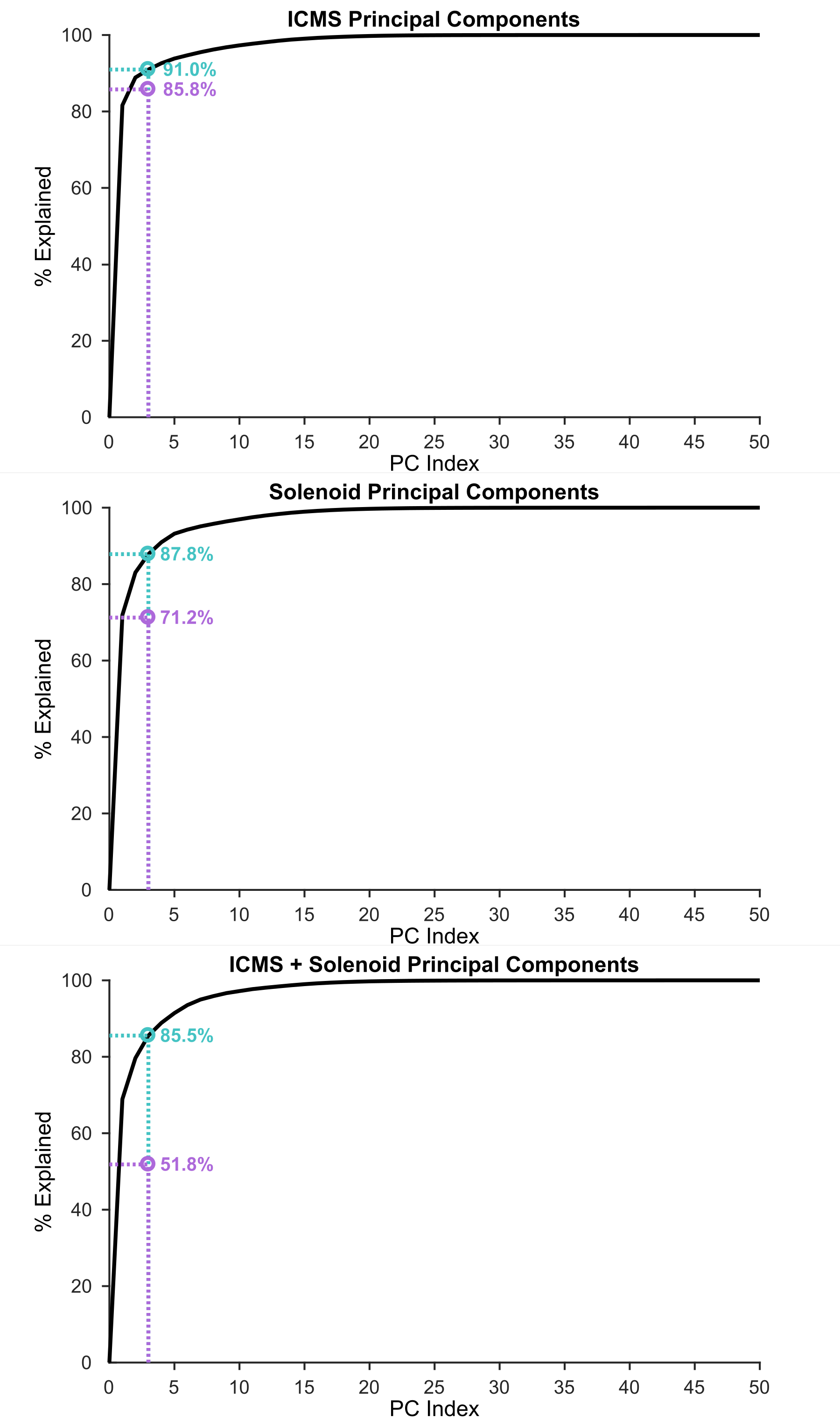

Supplement: Supplementary file 5 [file Image_5.TIF]

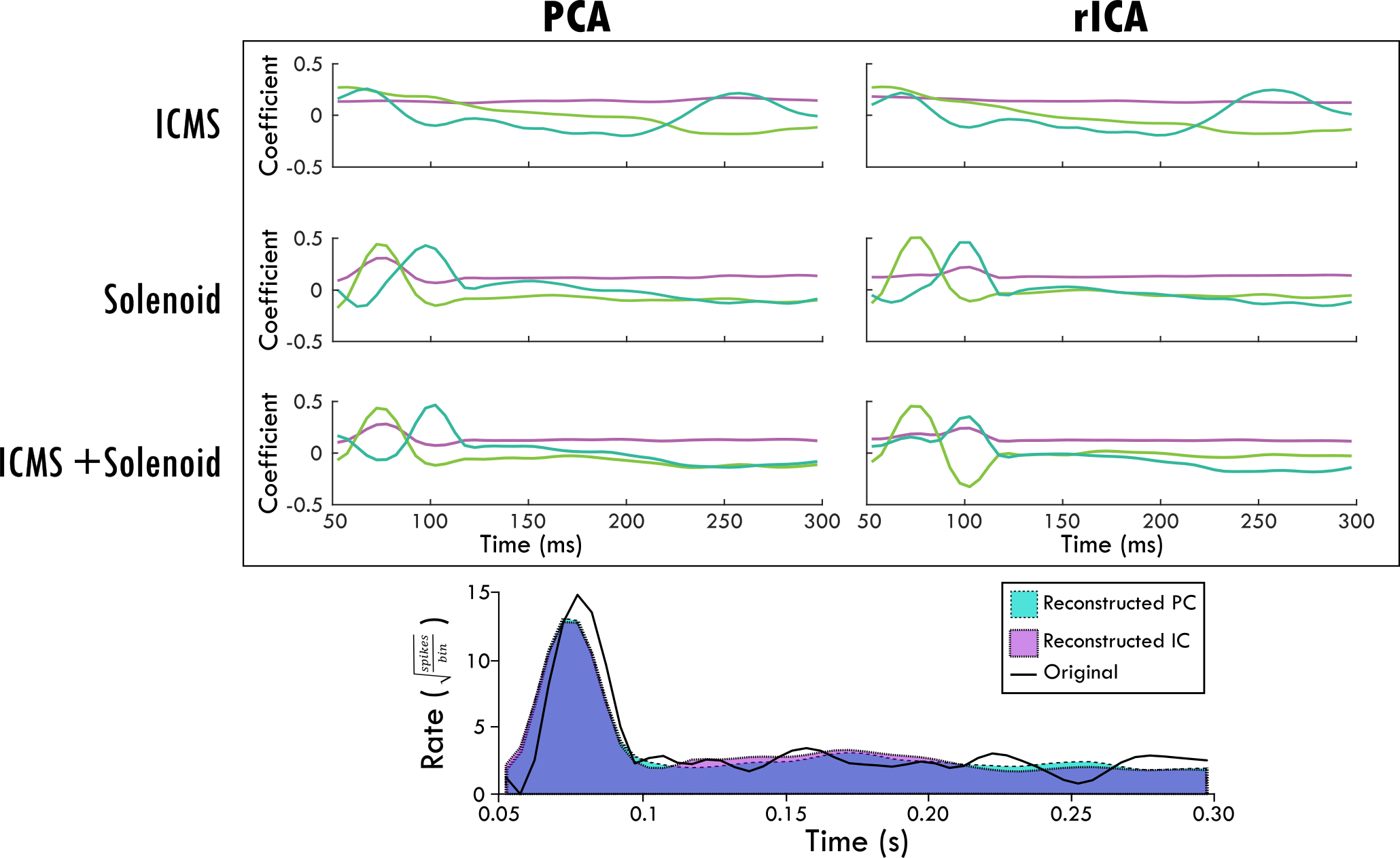

Supplement: Supplementary file 6 [file Image_6.TIF]
