# Supplementary material for: Post-ischemic reorganization of sensory responses in cerebral cortex
Source: Front Neurosci. 2023 Jun 2;17:1151309. doi: 10.3389/fnins.2023.1151309 (PMC10272353; doi:10.3389/fnins.2023.1151309)
Supplement: Supplementary file 7 [file Table_1.DOCX]

| **Animal** | **Lesion Volume**  **(mm^3^)** | **Standardized Scores** | | | |
| --- | --- | --- | --- | --- | --- |
|  |  | *Day 8* | *Day 12* | *Day 16* | *Day 20* |
| MM-W1 | 1.4 | -- | -3.2740 | 4.0961 | N/A |
| MM-U1 | 1.7 | 0.8578 | -0.2716 | 1.7121 | 5.4936 |
| MM-S1 | 3.1 | 5.2291 | 2.9809 | 3.7315 | 6.7804 |
| MM-V1 | 4.8 | -5.0754 | -7.4630 | -2.0480 | N/A |
| MM-T2 | 6.5 | -12.438 | -10.605 | -8.5496 | -0.2645 |
| MM-U2 | 8.8 | -3.6815 | -6.4801 | -1.8836 | 2.0838 |
| MM-T1 | 11.2 | -- | -20.777 | -- | -13.629 |

**Supplementary Table 1.** **Difference of each rat from baseline behavioral scores at each timepoint.** Standardized scores relative to a two-tailed T-distribution with a p value of 0.01 (t = 2.898, d.f. = 17) are highlighted (yellow values are below the confidence interval and gray those above). Timepoints with a dash represent days where the rat would not attempt reaches while N/A shows timepoints where the assay data is unavailable.
